# Influence of Temperature and Photoperiod on the Fecundity of *Habrobracon hebetor* Say (Hymenoptera: Braconidae) and on the Paralysis of Host Larvae, *Plodia interpunctella* (Hübner) (Lepidoptera: Pyralidae)

**DOI:** 10.3390/insects12080753

**Published:** 2021-08-20

**Authors:** George N. Mbata, Sanower Warsi, Mark E. Payton

**Affiliations:** 1 Agricultural Research Station, Fort Valley State University, Fort Valley, GA 31030, USA; szw0132@auburn.edu; 2 Department of Entomology and Plant Pathology, 301 Funchess Hall, Auburn University, Auburn, AL 36849, USA; 3 Department of Biomedical Sciences, Rocky Vista University, Parker, CO 80134, USA; mpayton@rvu.edu

**Keywords:** *Habrobracon hebetor*, *Plodia interpunctella*, fecundity, paralysis, biological control

## Abstract

**Simple Summary:**

This study illustrated the role of optimum temperatures of 25 and 30° in maximizing oviposition by the female *H. hebetor*. The optimum temperatures for paralysis of *P. interpunctella* larvae by *H. hebetor* were shown to be 28 and 30 °C at short exposure periods. However, at long exposure periods, the paralysis rates did not differ significantly. Photoperiod had no impact on oviposition or paralysis of *P. interpunctella* by the wasp.

**Abstract:**

Studies were carried out in the laboratory to understand the optimum environmental conditions at which the ectoparasitoid, *Habrobracon hebetor* Say (Hymenoptera: Braconidae), can paralyze and lay eggs when reared on the larvae of the stored product pest, *Plodia interpunctella* Hübner (Lepidoptera: Pyralidae). At the four temperatures investigated (20, 25, 30, and 35 °C), optimum temperatures for oviposition were found to be 25 and 30 °C, while 35 °C was the least favorable temperature. No significant differences were found between the percentages of diapausing and non-diapausing larvae paralyzed by the wasp at the temperatures of 20, 25, 30, 35 °C within 5 days. However, in another experiment that investigated the effect of photoperiods at different temperatures that included 15, 19 and 28 °C, the number of paralyzed larvae was highly reduced at low temperatures (15 °C) but photoperiods had no significant impact on the number of host larvae paralyzed. In addition, observations at short time intervals also showed that lower temperatures slowed down host larvae paralysis. The results suggest that *H. hebetor* can paralyze host larvae of *P. interpunctella* more efficiently and deposit more eggs at temperatures within the range of 20–30 °C.

## 1. Introduction

Several members of the Pyralidae family of the Lepidoptera constitute worldwide pests of postharvest commodities [1]. Larvae of these pyralid moths, particularly the Indian meal moth (*Plodia interpunctella* Hübner), infest raw commodities such as cereals, dried fruits, vegetables, peanuts, and value-added processed food such as wheat flour [2,3,4,5]. Additionally, frass and silk webbing of the larvae can mass commodities together, thereby accelerating the deterioration of stored produce [6,7]. The management of pyralid moths in warehouses, food processing plants, and in other storage structures depends on conventional chemical pesticides, some of which have been found to leave harmful residues in food and the environment [8,9,10,11]. In addition, most of the conventional pesticides are facing elimination or restricted use [12,13].

Food safety and environmental concerns are among the factors prompting postharvest pest management experts and warehouse managers to search for safe non-toxic and sustainable alternative approaches that can overcome the challenges caused by insect pests [14]. Parasitic wasps represent an alternative and environmentally friendly approach in postharvest systems for the management of pest populations because parasitoids are environmentally safe and do not negatively impact humans or beneficial organisms [14]. *Habrobracon hebetor* Say (Hymenoptera: Braconidae) is an important ectoparasitoid that has already been demonstrated to have biocontrol potentials and is able to regulate a wide range of stored product moth pests including *P. interpunctella* [15]. An additional advantage of parasitic wasps is that in some cases, applications of these in postharvest integrated pest management (IPM) reduced insect parts and fragments in commodity [16]. However, rearing large numbers for inundative or inoculative releases in pest management programs has been challenging due to the biology of parasitoid and its host species [17,18].

One of the major challenges in rearing large number of *H. hebetor* is the narrow window of approximately 5 days that the female wasp has to paralyze the host, lay eggs and develop on the host pyralid moth [19]. The 4th and 5th host larvae are the suitable stages for the development of the wasp and these larval stages pupate within days and become unpalatable to the female wasp [19]. Other factors that are likely to enhance the oviposition and development of *H. hebetor* include favorable environmental factors, such as temperature and photoperiods.

Previous studies have examined the effects of temperature on the oviposition, development and parasitism efficiency of *H. hebetor* [20,21,22]. *Habrobracon hebetor* laid eggs at the temperature range from 16 to 44 °C but 30 °C was the optimum temperature for the maximum egg laying [23]. Similarly, it has been observed that the net fecundity rate and total fecundity rate of *H. hebetor* were high at 30 °C compared to 20 and 40 °C [24]. In addition, the maximum number of *H. hebetor* offspring emerged at 30 °C followed by 40 and 20 °C [10]. *Habrobracon hebetor* laid the maximum number of eggs on the 3rd and 5th day of oviposition when fed on diapausing and non-diapausing larvae of *P. interpunctella*, respectively, at 28 °C and with a 16:8 LD cycle [14]. Additionally, *H. hebetor* had a 0.184, 0.215, 0.261 and 0.234 innate capacity of increase (r_m_) values at 20, 22, 30 and 40 °C, respectively [24,25]. Host parasitism by *H. hebetor* has been shown to vary with the temperature and the level of parasitism sharply decreased with increased temperature [22]. *Habrobracon hebetor* has been demonstrated to prefer diapausing than non-diapausing larvae for oviposition and development, and a female wasp deposits a mean of 17.46 and 9.64 eggs per day on diapausing and non-diapausing last instars of *P. interpunctella,* respectively [14,15]. However, there is a dearth of information on how combinations of temperature and photoperiod will impact paralysis of *P. interpunctella* larvae by *H. hebetor* and oviposition by the female wasp.

The current study investigated paralysis of the host, *P. interpunctella* larvae, by the wasp, *H. hebetor,* and oviposition by the wasp at combinations of different temperatures and photoperiods for 48, 24 h and at shorter exposure periods.

## 2. Material and Methods

### 2.1. Insect Rearing (Plodia interpunctella and Habrobracon hebetor) in the Laboratory

Initial cultures of the *P. interpunctella* and *H. hebetor* used in this study were collected in 2000 and 2001, respectively, from the United States Department of Agriculture, Agriculture Research Station (USDA, ARS) Center for Grain and Animal Health Research (CGAHR), Manhattan, KS 66502, USA, and had since been maintained at Fort Valley State University (FVSU) rearing facility. The laboratory populations of *P. interpunctella* and *H. hebetor* had been augmented periodically with field collected moths and wasps, respectively, to avoid problems associated with prolonged laboratory breeding. The artificial diet used to rear the moth was formulated with corn meal, chick starter mash, oats, glycerol and yeast at a volumetric ratio of 4:2:2:1:0.1 [26]. Rearing jars (1 L) containing *P. interpunctella* larvae were kept in chambers maintained at 28 ± 1.5 °C 16L:8D (photoperiod) and 70 ± 5% RH to become fully grown, non-diapausing last instar [15]. Ten-d-old (3rd instar) non diapausing larvae were transferred to a chamber maintained at a temperature of 14 ± 1.5 °C, 10L:14D (photoperiod) to induce diapause in the larvae [27]. For these experiments, 40-d-old diapausing and 18-d-old non-diapausing 5th instar were used.

The adult parasitoids were fed on fully-grown larvae of *P. interpunctella* at 28 ± 1.5 °C, 1L6:8D (photoperiod) and 70 ± 5% RH and the generation time was approximately 20 d [26].

### 2.2. Effect of Temperature on Oviposition of H. hebetor Provisioned with Diapausing and Non-Diapausing P. interpunctella Larvae

The effect of temperature on the oviposition by *H. hebetor* provisioned with diapausing and non-diapausing larvae was investigated at 20, 25, 30 and 35 (±0.5) °C, 70 (±1.5)% RH, 16L: 8D (photoperiod) in cooled incubators, Percival I-36 (Percival, 505 Research Dr., Perry, IA 50220, USA). Oviposition was assessed by counting the number of eggs laid by each female parasitoid wasp under a dissecting microscope. At each temperature, two sets of 5 glass jars (1000 mL) were prepared, a set with 2 diapausing larvae per jar and the second set with 2 non-diapausing larvae per jar. A pair (2 d old, male and female) of *H. hebetor* was added to each jar. The jars containing *P. interpunctella* larvae and wasps were transferred to incubators maintained at 20, 25, 30 and 35 (±0.5) °C, 70 (±1.5)% RH, 16L: 8D (photoperiod). Every 24 h, the parasitoids were transferred to new jars containing fresh host larvae until the female parasitoid died, while the old jars and host larvae were examined and parasitoid eggs counted. Three trials were carried out with three generations of the wasp and host *P. interpunctella*.

### 2.3. Effect of Temperature on the Paralysis of Diapausing and Non-Diapausing Larvae of P. interpunctella by H. hebetor

The effect of temperature on the ability of *H. hebetor* to paralyze diapausing and non-diapausing *P. interpunctella* larvae was investigated at 20, 25, 30 and 35 (±0.5) °C; 70 (±1.5) % RH, 16L: 8D (photoperiod). Two sets of 5 jars (1000 mL) each containing 10 host larvae and a pair (2 d old, male and female) of *H. hebetor* were set up, one set for diapausing larvae and the second set for non-diapausing larvae at each of the temperatures investigated. The jars were transferred to incubators maintained at the desired temperatures (20, 25, 30 and 35 °C) for 5 d. Thereafter, paralyzed host larvae were enumerated and recorded. Paralysis in host larvae was induced by the female wasp injecting venom into the host larvae. Following paralysis, the host larvae became numb and unable to respond to touch with sharp forceps. Three trials were carried out with three different generations of parasitoid and host *P. interpunctella* larvae.

### 2.4. Effect of Temperature and Photoperiod on Host Larvae Paralysis within 24 and 48 h Periods

The combined effect of temperature and photoperiod on host larvae paralysis caused by the wasp was investigated for non-diapausing larvae of *P. interpunctella* at 15, 19 and 28 (±0.5) °C and three light regimes; alternate light and dark cycle (12L:12D), continuous light (24 LL) and continuous darkness (24 DD). Five jars (1000 mL) were set up at each of the 18 treatments. Ten host larvae and one pair of *H. hebetor* (2 d old, male and female) were placed in each of 1000 mL rearing jars. A set of 30 jars dispensed with 10 host larvae and 1 pair of wasps each was set up at each temperature tested. The jars containing host larvae and wasps were placed in incubators maintained at 15, 19 and 28 °C under 12 h L: 12 h D, 24 h LL and 24 h DD photoperiods and 70 ± 5% RH in incubators. The distribution of the jars is as follows; at 15 °C, 10 jars each were placed at LL, LD and DD chambers. Paralyzed host larvae in 5 jars at each set of combinations (example 15 °C, LL) were enumerated at 24 h and remaining 5 jars had paralyzed larvae counted after 48 h. Three trials were carried out with different generations of the wasps.

### 2.5. Effect of Temperature on the Paralysis of P. interpunctella Larvae by H. hebetor at Short-Term Exposure Periods (1, 2, 3, 6 h) under Light or Dark Conditions

Sets of 1000 mL jars were dispensed with 10 fully grown larvae of *P. interpunctella* and a pair (male and female) *H. hebetor* each. Two sub-sets of 4 jars were transferred to incubators maintained at 14, 20, 28, 30 and 35 (±0.5) °C, and at either light or dark condition. A jar was withdrawn from each of the incubators after 1, 2, 3, or 6 h and the number of paralyzed larvae was counted. A total of 10 trials were carried out with three parasitoid generations.

### 2.6. Statistical Analysis

The variables examined included percentage host larvae paralyzed and number of eggs deposited by the parasitoid. The independent variables in this study were temperature, photoperiods, and larval types (diapausing and non-diapausing host larvae). Data were analyzed using Statistical Analysis System version 9.4 [28]. All percentage data were arcsine of square root transformed before analyses to minimize variances and standardize means [29]. Treatments were constructed as combinations of factors, and simple effects of these factors were analyzed with planned contrasts. The Satterthwaite approximation was used in determining DF for the effect of temperature on lifetime oviposition of wasp provisioned with diapausing and non-diapausing larvae. For the data on the effect of temperature and photoperiod on the paralysis of host larvae at short-term exposure periods, a repeated-measures analysis was conducted using autoregressive period 1 covariance structure. Raw mean percentages and standard errors are reported. Statistical significance was determined by *p* < 0.05.

## 3. Results

### 3.1. Effect of Temperature on the Lifetime Oviposition of H. hebetor Provisioned with Diapausing or Non-Diapausing P. interpunctella Larvae

The numbers of eggs laid by mated female *H. hebetor* depended on the host larval type, diapausing or non-diapausing, and temperature, and these two factors significantly interacted with each other (Table 1 and Table 2). Females provisioned with diapausing host larvae laid more eggs than those provisioned with non-diapausing larvae. The highest numbers of eggs were laid at 30 °C, and egg laying by the wasp was significantly better at 20 and 25 °C than at 35 °C (Table 1 and Table 2).

### 3.2. Effect of Temperature on of Paralysis of Diapausing and Non-Diapausing Larvae of P. interpunctella by H. hebetor

The percentages of both diapausing and non-diapausing larvae paralyzed at all the temperatures investigated ranged between 85.3% and 96.8% and were not statistically different (Table 3 and Table 4*)*. A five-day period was enough time for nearly all the host larvae to be paralyzed at the host density of the current study.

### 3.3. Interaction of Temperature and Photoperiod on the Paralysis of Host Larvae by H. hebetor within 24 and 48 h Periods

Temperature had a significant effect on the paralysis of host larvae and the highest percentage of paralyzed host larvae was observed at 28 °C, while the lowest was at 15 °C (Table 5 and Table 6). However, photoperiod did not have any impact on the ability of *H. hebetor* to paralyze *P. interpunctella* larvae (Table 5 and Table 6). Longer exposure period of 48 h favored paralysis of the host larvae by the female wasp as the percentages of paralyzed larvae at 48 h were significantly higher than those paralyzed within 24 h period (Table 5 and Table 6). Statistical analysis did not show any interaction between photoperiod and temperature (Table 5 and Table 6).

### 3.4. Effect of Temperature on the Paralysis of P. interpunctella Larvae by H. hebetor at Short-Term Exposure Periods (1, 2, 3, 6 h) under Light or Dark Conditions

Temperature had a significant effect on the ability of *H. hebetor* to paralyze the host larvae at short exposure periods (Table 7 and Table 8). Moderately high temperatures (28 and 30 °C) favored paralysis of host larvae by the wasp compared with low temperatures (14, 20 °C) and high temperature (35 °C). Dark and light conditions did not have effect on the percentage of host larvae paralyzed by the wasp but there was a significant interaction between temperature and light/dark conditions (Table 7 and Table 8). The percentages of paralyzed host larvae increased with the duration of exposure of host larvae to wasps (Table 7 and Table 8). The least percentage of paralyzed host larvae by the wasps occurred after 1 h exposure period, while the highest percentages of paralyzed host larvae were at 6 h exposure periods at both light and dark conditions (*p* < 0.05; N = 400; Table 7 and Table 8).

## 4. Discussion

The temperatures at which the highest numbers of eggs were laid by female *H. hebetor* in the current study were 25 and 30 °C. In a temperature-dependent study of *H. hebetor* reared on larvae of *Galleria mellonella* (L.), it was found that temperature range between 25 and 32 °C favored mass rearing of *H. hebetor* [30]. It is probable that the low progeny production by *H. hebetor* observed outside the optimum temperature range [30] could be due to low oviposition by *H. hebetor* as was observed at 20 and 35 °C in the current study. Reduction in oviposition was observed for wasps provisioned with diapausing or non-diapausing host larvae at temperatures outside the optimal range of 20–32 °C. However, the number of eggs laid by wasps provisioned with diapausing host larvae was still higher compared with the number of eggs laid by wasps provisioned with non-diapausing larvae. These results confirm previous observations on the improved oviposition by *H. hebetor* females provisioned with diapausing *H. hebetor* [14,15].

At temperatures that ranged between 20 and 35 °C, *H. hebetor* females paralyzed between 85.2 and 96.8% host larvae, and these were not significantly different. However, at short exposure periods (1, 2, 3 and 6 h), percentages of larvae paralyzed were significantly different, with more host larvae paralyzed earlier at 28 and 30 °C than at other temperatures that were investigated. Temperatures outside the optimum range of 25 through 32 °C were less favorable for host larvae paralysis [30]. Previous studies have documented highest paralyzing efficiency (~99%) at 25 °C [22] and low host larvae paralysis at 35 °C [20]. In addition, *H. hebetor* high attack rates have been observed at 20, 25 and 30 °C [21]. High temperatures are likely to cause host larvae to produce a lot of webbing that could shield the host larvae from being paralyzed by the wasps, while low temperatures could slow down movements by the wasp resulting in reduced attacks on host larvae [15]. Since non-optimal low and high temperatures have been demonstrated to reduce rates of paralysis and oviposition by the *H. hebetor*, is it possible that a delay in host larvae paralysis could reduce oviposition potential of *H. hebetor*? The impact of promptness of host larvae paralysis on the oviposition potential of *H. hebetor* should be investigated.

Photoperiods did not impact the ability of wasps to paralyze host larvae at both long term (24 and 48 h) and short-term exposure periods. The results suggest that diurnal rhythm may not play a role in the parasitism of host larvae by *H. hebetor.* In addition, visual stimuli may not be among the cues that elicit attack or stinging of the host larvae by *H. hebetor*. Volatile cues emanating from hosts and host habitat have been suggested to be the more likely stimuli that elicit attraction of *H. hebetor* to pyralid larval hosts [31,32,33].

## Figures and Tables

**Table 1:** Effect of temperatures on lifetime oviposition of *H. hebetor* provisioned with diapausing or non-diapausing host larvae of *P. interpunctella.*

Temperature (°C)	Mean Number of Eggs Laid by Parasitoids (±S.E) (n = Treatment Sample Size)	
	Diapausing Larvae	Non-Diapausing Larvae
20	72.2 ± 4.1 A b * (15)	34.0 ± 3.2 B c (15)
25	98.4 ± 6.5 A ** a b (15)	68.0 ± 3.9 B b (15)
30	120.0 ± 5.3 A a (15)	80.8 ± 3.4 B a (15)
35	41.0 ± 2.2 A c (15)	17.6 ± 1.5 B c (15)

* Means within a column followed by the same lowercase letter are not significantly different (*p* < 0.05); ** means within a row with the same uppercase letter are not significantly different (*p* < 0.05); mean separation was by the Tukey–Kramer HSD mean comparison procedure at α = 0.05.

**Table 2:** ANOVA results for the main effects of temperature and host larvae type (diapausing and non-diapausing host larvae) of *Plodia interpunctella* on the fecundity of *Habrobracon hebetor.*

ANOVA	*p* Value	DF	F Value
Larval type	<0.0001	1, 84.3	57.01
Temperature	0.0001	3, 81.2	81.10
Larval type × Temperature	0.0009	3, 81.9	6.08

DFs were based on the Satterthwaite approximation; significance level at 5%.

**Table 3:** Effect of temperature on the paralysis of *P. interpunctella* by mated female wasp.

Temperature (°C)	Host Paralyzed (Means ± S.E) (n = Treatment Sample Size)	
	Diapausing Larvae	Non-Diapausing Larvae
20	93.9 ± 2.4 a * A (15)	90.6 ± 1.5 a A (15)
25	96.8 ± 1.8 a A ** (15)	96.4 ± 1.4 a A (15)
30	89.8 ± 2.0 a A (15)	85.2 ± 1.7 a A (15)
35	85.3 ± 3.0 a A (15)	85.5 ± 1.5 a A (15)

* Means within a column followed by the same lowercase letter are not significantly different using the Tukey–Kramer HSD mean comparison procedure at α = 0.05. ** Means within a row followed by the same uppercase letter are not significantly different using the Tukey–Kramer HSD mean comparison procedure at α = 0.05.

**Table 4:** ANOVA results for the main effects and interactions of type of larvae (diapausing and non-diapausing host larvae) and photoperiods at 19 °C on host mortality and parasitoid progeny production.

Variables	Host Mortality		
Source	**DF**	**F**	***p***
Larval type	1, 112	2.51	0.06
Temperature	3, 112	19.30	0.09
Larval type × temperature	3, 112	1.00	0.3941

**Table 5:** Influence of photoperiod and temperatures on the paralysis of host larvae by *H. hebetor* after 24 and 48 h.

Photoperiod (h)	Mean % Host Mortality (±S.E.) (n = Treatment Sample Size)					
	15 °C		19 °C		28 °C	
	24 h	48 h	24 h	48 h	24 h	48 h
12L: 12L	21.0 ± 2.6 a * D (15)	61.0 ± 4.5 a B (15)	33.0 ± 3.7 a D (15)	74.0 ± 4.4 a A B (15)	47.0 ± 4.7 b C (15)	86.0 ± 3.5 a A (15)
12L: 12D	27.0 ± 3.4 a D ** (15)	63.0 ± 3.9 a B (15)	46.0 ± 5.0 a C (15)	84.0 ± 3.0 a A (15)	55.0 ± 4.8 a b C (15)	97.0 ± 1.7 a A (15)
12D: 12D	21.0 ± 3.1 a E (15)	68.0 ± 4.7 a B C (15)	34.0 ± 4.7 a D (15)	76.0 ± 4.3 a B (15)	61.0 ± 4.4 a C (15)	98.0 ± 4.3 a A (15)

* Means within a column followed by the same lowercase letter are not significantly different using the Tukey–Kramer HSD mean comparison procedure at α = 0.05. ** Means within a row followed by the same uppercase letter are not significantly different using the Tukey–Kramer HSD mean comparison procedure at α = 0.05.

**Table 6:** ANOVA results for the main effects and interactions of photoperiod and temperatures on the paralysis of host larvae by *H. hebetor* after 24 and 48 h.

Source	DF *	F	*p*
Temperature	2, 238	391.68	<0.001
Photoperiod	2, 238	2.51	0.09
Exposure Period (EP)	1, 238	1645.82	<0.0001
Temperature × Photoperiod	4, 238	3.09	0.07
EP × Photoperiod	2, 238	3.70	0.0263
Temperature × EP	4, 238	15.39	<0.0001
Temperature × EP × Photoperiod	4, 238	3.29	<0.0119

* DF for replication = 14.

**Table 7:** Short-term (1, 2, 3 or 6 h) effect of temperature and photoperiod on the paralysis of *P. interpunctella* larvae exposed to *H. hebetor*.

Temperature (°C)	Mean % Host Mortality (±SE) (n = Treatment Sample Size)							
	Light Condition				Dark Condition			
	Host Exposure Time (h)				Host Exposure Time (h)			
	One	Two	Three	Six	One	Two	Three	Six
14	5.0 ± 2.2 C c * (10)	5.0 ± 2.2 C c (10)	13.0 ± 3.4 C c (10)	48.0 ± 3.2 A b (10)	5.0 ± 2.2 C c (10)	6.0 ± 2.4 C c (10)	28.0 ± 4.8 B b (10)	43.0 ± 5.0 A b (10)
20	10.0 ± 3. D b c (10)	19.0 ± 4.0 D b (10)	33.0 ± 4.7 C b (10)	65.0 ± 2.8 A a (10)	10 ± 3.00 D b c (10)	19.0 ± 3.92 D b (10)	34 ± 4.73 C b (10)	57.0 ± 4.7 B a (10)
28	15.0 ± 3.5 E a b (10)	35.0 ± 4.7 C a (10)	54.0 ± 4.9 B C a (10)	71.0 ± 3.5 A a (10)	15.0 ± 3.5 E a b c (10)	35.0 ± 4.7 D a (10)	47.0 ± 4.9 C a (10)	61.0 ± 4.5 B a (10)
30	21.0 ± 4.1 C a (10)	35.0 ± 4.7 C a (10)	59.0 ± 4.9 A a (10)	66.0 ± 1.9 A a (10)	21.0 ± 4.1 C a (10)	32.0 ± 4.6 C a (10)	44.0 ± 4.7 B a b (10)	64.0 ± 4.3 A a (10)
35	5.0 ± 2.1 D ** c (10)	6.0 ± 2.3 D c (10)	48.0 ± 4.7 B a b (10)	52.0 ± 4.9 A b (10)	10.0 ± 4.0 D b c (10)	25.0 ± 4.7 C a b (10)	39.0 ± 4.8 B a b (10)	56.0 ± 3.9 A B a (10)

* Means within a column above followed by the same lowercase letter are not significantly different. ** Means across a row above followed by the same uppercase letter are not significantly different. The Tukey–Kramer HSD means comparison procedure at significance level *p* ≤ 0.05.

**Table 8:** ANOVA results for the main effects and interactions of short-term (1, 2, 3 or 6 h) effect of temperature and photoperiod on the paralysis of *P. interpunctella* larvae exposed to *H. hebetor.*

	DF	F Value	*p* Value
Temperature	4, 39	90.22	<0.0001
Photoperiod (Light/Dark)	1, 39	0.97	0.3313
Exposure Period	4, 39	1.36	0.2557
Temperature × Photoperiod	12, 39	5.81	<0.0001
Temperature × Exposure Period	12, 39	5.81	<0.0001
Photoperiod × Exposure Period	3, 39	20.38	<0.0001
Temperature × Photoperiod × Exposure Period	12, 39	1.92	0.0622

## Data Availability

Data can be provided on request from the lead author.
